# Prognosis of COVID-19 in Patients with Liver and Kidney Diseases: An Early Systematic Review and Meta-Analysis

**DOI:** 10.3390/tropicalmed5020080

**Published:** 2020-05-15

**Authors:** Tope Oyelade, Jaber Alqahtani, Gabriele Canciani

**Affiliations:** 1 Institute for Liver and Digestive Health, Division of Medicine, University College London, London NW3 2PF, UK; gabriele.canciani20@gmail.com; 2 School of Respiratory Medicine, University College London, London NW3 2PF, UK; jaber.alqahtani.18@ucl.ac.uk; 3 School of Medicine, La Sapienza University, Rome 00185, Italy

**Keywords:** COVID-19, SARS-CoV-2, hepatitis B and C, cirrhosis, chronic kidney disease, alcohol-related liver disease, nonalcoholic steatohepatitis, necrosis

## Abstract

The mortality and severity in COVID-19 is increased in patients with comorbidities. The aim of this study was to evaluate the severity and mortality in COVID-19 patients with underlying kidney and liver diseases. We retrieved data on the clinical features and primary composite end point of COVID-19 patients from Medline and Embase which had been released from inception by the April 16, 2020. The data on two comorbidities, liver diseases and chronic kidney disease, were pooled and statistically analysed to explain the associated severity and mortality rate. One hundred and forty-two abstracts were screened, and 41 full articles were then read. In total, 22 studies including 5595 COVID-19 patients were included in this study with case fatality rate of 16%. The prevalence of liver diseases and chronic kidney disease (CKD) were 3% (95% CI; 2–3%) and 1% (95% CI; 1–2%), respectively. In patients with COVID-19 and underlying liver diseases, 57.33% (43/75) of cases were severe, with 17.65% mortality, while in CKD patients, 83.93% (47/56) of cases were severe and 53.33% (8/15) mortality was reported. This study found an increased risk of severity and mortality in COVID-19 patients with liver diseases or CKD. This will lead to better clinical management and inform the process of implementing more stringent preventative measures for this group of patients.

## 1. Introduction

The 2010 Global Burden of Disease reported that liver diseases were responsible for about 2 million deaths annually, with 50% of these associated with complications due to liver cirrhosis and the other half linked to hepatocellular carcinoma and viral hepatitis [1]. Cirrhosis is an end stage of chronic liver disease often preceded by hepatocellular necrosis and progressive fibrosis triggered by various agents including viral infections and chronic alcohol use [2]. Alcohol-related liver disease, nonalcoholic steatohepatitis and hepatitis B and C have been reported to be the main aetiologies of liver cirrhosis, with an up to 80% mortality rate recorded 1-year after decompensation [3,4]. Aside from mortality, the economic impact of liver-associated morbidity is also high, with associated disease-adjusted life years loss at over 41 million years globally. According to the World Health Organization (WHO) global health estimate of 2015, chronic liver disease ranks as the 16th highest cause of morbidity globally [5,6]. Despite the availability of vaccines for hepatitis B and the advances in clinical understanding and the management of chronic liver diseases, the global health burden of the disease increased between 1990 and 2017. This rise in health burden was attributed to ageing and an overall increase in the global population [4].

According to Kidney Disease Improving Global Outcome (KDIGO), chronic kidney disease (CKD) is a dysfunction of the kidney characterised by established histological damage or a suboptimal (<60 mL/min/1.73 m^2^) glomerular filtration rate (GFR) persisting for at least 3 months [7]. Although the majority of CKD cases are linked to diabetes and hypertension [8], other risk factors, including genetics [9], recreational drugs and alcohol consumption [10], obesity [11], gender [12,13], age [12], lower birth weight [14], smoking status [15,16], ethnicity [17], family history of CKD [18] and acute kidney injury, have been studied [19,20]. In 2017, the number of deaths associated with CKD or CDK-related complications was estimated to be 1.2 million, accounting for 4.6% of global deaths [21]. Between 1990 and 2017, CKD rose as a cause of global mortality from the 17th to the 12th leading cause of death, with a 46% increase in the total number of deaths caused directly or indirectly by cardiovascular disease linked to kidney dysfunction [22]. While the relationship between COVID-19-induced acute kidney injury has been investigated previously [23], to the best of our knowledge, no studies have looked at the risk of COVID-19 in patients with all-form renal disease.

The severe acute respiratory syndrome coronavirus 2 (SARS-CoV-2) is a viral pathogen which is responsible for the coronavirus disease 2019 (COVID-19) [24]. Symptoms of COVID-19 include fever, fatigue, dry cough, dyspnoea and sore throat, with patients presenting with abnormal chest CT (Computed Tomography) scans in the form of pulmonary ground glass opacity changes [25,26]. COVID-19 was first reported in December 2019, with its possible origin linked to the Wuhan seafood market in China [27]. Since first being reported, SARS-CoV-2 has infected, as of 2nd of April, 2020, 896,450 people and caused 45,525 deaths worldwide, with these numbers rising daily [28]. So far, the risk factors associated with poor clinical outcomes (death or admission to an intensive care unit (ICU)) have been reported to be old age and several comorbidities associated with compromised immune system to help the patient fight the infection. The most common of these comorbidities are hypertension, diabetes, cardiovascular diseases and malignancies. These comorbidities, individually or in combination with age, were reported to be linked with poor prognoses [29]. Several studies have looked at the risk posed to patients with various chronic diseases by COVID-19. For instance, Alqahtani et al. 2020 looked at the risk of smoking status and chronic obstructive pulmonary disease (COPD) in COVID-19 patients, establishing an increased risk of death or admission to ICU for patients with COPD or smoking history infected with SARS-CoV-2 [30]. 

While COVID-19-induced liver and kidney injuries have been documented, to the best of our knowledge, there has been no report on the risk posed by COVID-19 infection in patients with a history of liver or renal disease. Understanding the risk to this subpopulation of patients will facilitate effective prevention decisions and clinical management. We aim here to understand the risks by looking at reported cases since the outbreak of COVID-19. 

## 2. Methods

Preferred Reporting in Systematic Reviews and Meta-Analyses (PRISMA) guidelines were followed during the drafting of this review. We searched Medline and Embase from November 2019 to 10 April 2020 and later, on the 14 April 2020, an updated search was performed. The search strategy was designed to include all papers on COVID-19 published from 21 November 2019, when the first case of the disease was reported, up to right before this review was submitted (Table A1 in Appendix A).

### 2.1. Inclusion and Exclusion Criteria

Studies considered were those reporting the clinical characteristics of diagnosed COVID-19 patients with underlying kidney and/or liver diseases. To be eligible, studies had to also report clinical outcome in the form of disease severity (defined as admission to ICU or need for a respirator or intubation) as well as death. Excluded studies were those including COVID-19 patient clinical features but not liver or kidney diseases comorbidity, SARS (Severe Acute Respiratory Syndrome), MERS (Middle East Respiratory Syndrome) and other coronavirus infections, non-English manuscripts, reviews, qualitative studies, editorials and letters of correspondence.

### 2.2. Data Collection

Potential studies were initially screened by two of the authors (T.O. and J.A.), who scrutinised the title and abstract and came to a final decision on whether each study should be included. Included studies were then fully read by the authors to identify which of the included studies satisfied the inclusion criteria stated above. The references of the finally selected studies were then screened for other eligible studies. A third author was consulted throughout the selection period to resolve conflicts between the two authors. Selected reports were uploaded to Endnote, and duplicates were removed. The duplicate-free studies were uploaded to the Rayyan review software for screening based on title, abstract and then full text by two independent reviewers.

### 2.3. Quality Assessment

The quality of the included studies was independently assessed by two authors using a modified version of the Newcastle-Ottawa Scale (NOS) [31]. Accordingly, the modified NOS included three domains and six questions scored with a star if satisfied and no star if otherwise. The domains covered assessments of the quality of “Selection”, “Ascertainment” and “Outcome”. The “Selection” domain describes the adequacy of the sample sizes and representativeness of the study population. A sample size of ≥29 patients was considered adequate, as it represents the lower range of the included studies where clinical characteristics and outcomes were adequately presented. Studies including multiple centres were given an extra point. The “Ascertainment” domain evaluated the adequacy of the confirmatory test and mode of recording comorbidity. The use of polymerase chain reaction (PCR) tests for diagnosis, as recommended by the WHO [32], was scored, as well as the use of electronic medical records (EMRs) to confirm comorbidities. Verbal confirmation of comorbidities was considered inadequate. The “Outcome” domain scored the adequacy of how outcomes were reported and the follow-up period. Outcomes reported by qualified clinical staff and a follow-up of at least two weeks, as recommended by the European Centre for Disease Prevention and Control (ECDC), were scored (Table A1).

### 2.4. Data Extraction and Analysis

**Figure 1:**
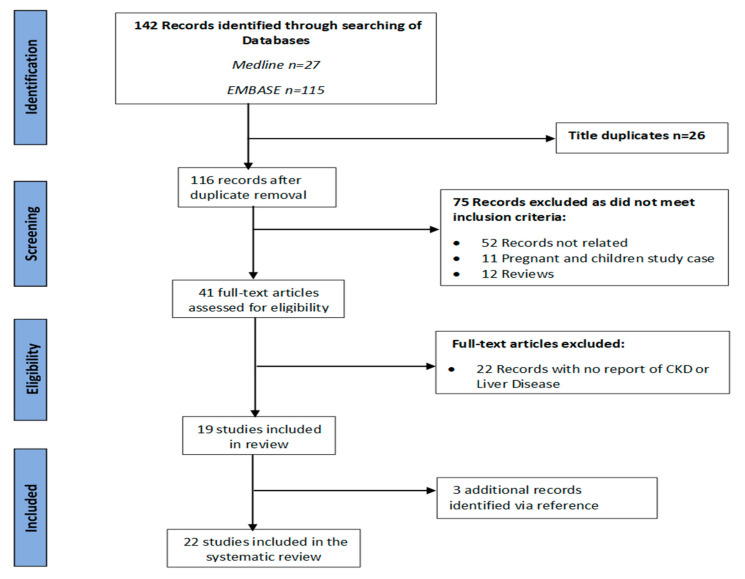
Risk of COVID-19 in patients with chronic liver and kidney diseases: a systematic review according to the Preferred Reporting Items for Systematic Reviews and Metanalyses diagram.

**Figure 2:**
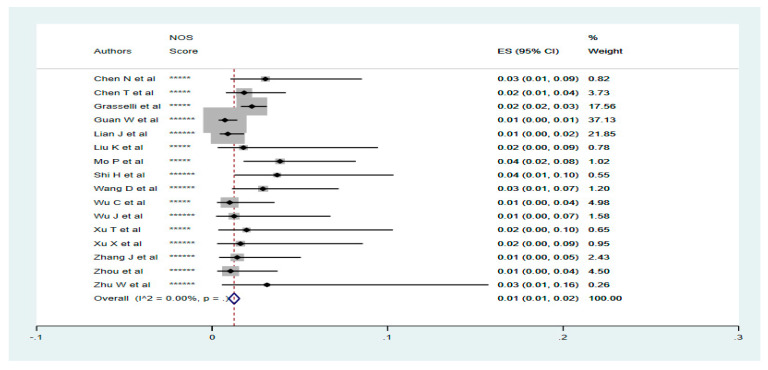
Pooled prevalence of patients with chronic renal diseases diagnosed with COVID-19. The red dotted line represents the overall effect size of the studies (0.01). The lateral edges of the blue diamond represent the limits of the 95% confidence intervals (0.01, 0.02). ES = Effect Size, NOS = Newcastle-Ottawa Score.

All analyses were performed using the Stata/SE15 software. The pooled prevalence of patients with CKD and liver diseases was analysed using the Metaprop procedure in Stata. Fixed and random effect models were used depending on the level of heterogeneity observed between the included studies. Forest plots were generated presenting the effect sizes (95% CI), percentage weights and the between-studies heterogeneity (I^2^ Statistic, *p*-value, Figure 1 and Figure 2). The prevalence and clinical outcomes of COVID-19 patients with CKD and liver diseases were synthesised from all included studies. Primary composite end points were disease severity and mortality. Disease severity was defined as extended hospital stay, admission to ICU or need for mechanical ventilation.

## 3. Results

The initial database search generated 142 papers, from which 26 duplicates were removed. After the title and abstract were screened, 75 papers were excluded and 41 included based on the inclusion criteria. After the full-text reviews, another 22 studies were excluded, resulting in 19 studies with the desired criteria. All the references of the 19 included studies were then screened for studies relevant to the review; three more studies were included from the references, making a total of 22 studies (Figure 1). The modified NOS assessment performed showed a low risk of bias in the included studies (Table A1).

### 3.1. Description of Included Studies

The total number of confirmed COVID-19 cases included in this study was 5595, of which 2045 (36.55%) were female. Where reported, 147/5305 (2.77%) and 83/5038 (1.65%) had comorbidities of liver diseases and CKD respectively. The mean ±SD (range) of the sample sizes of all included studies was 254.32 ± 385.76 (29–1591). One of the studies was conducted in Italy and the rest in China. Fifteen of the 22 studies, comprising 4367 patients, reported mortality. Where reported, the mortality was 710/4367 (16.26%) in this review. The mean (±SD) follow-up time was 30.55 ± 13.24 days. The clinical characteristics of the liver diseases and CKD, including the stages and aetiology, were not provided in all the studies (Table 1).

### 3.2. Prevalence of Renal Diseases in Confirmed COVID-19 Cases

The prevalence of CKDs in patients diagnosed with COVID-19 was 1% (95% CI; 1–2%). A random effect model was initially used to pool the studies. However, this was changed to the fixed effect model because of the observed low level of between-studies heterogeneity (I^2^ = 27.60%, *p* = 0.15; Data not shown). 

### 3.3. Disease Outcome for Renal Diseases Patients with COVID-19

In all, 5 studies including 3123 COVID-19 patients, 56 of which had CKD, reported severity. Where reported, the severity of COVID-19 was 83.93% (47/56) in patients with underlying CKD. Only 3 studies, including 15 COVID-19 patients with CKD, reported mortality. The mortality in patients with CKD diagnosed with COVID-19 was 53.33% (8/15) (Table 1).

### 3.4. Prevalence of Liver Diseases in Confirmed COVID-19 Cases

**Figure 3:**
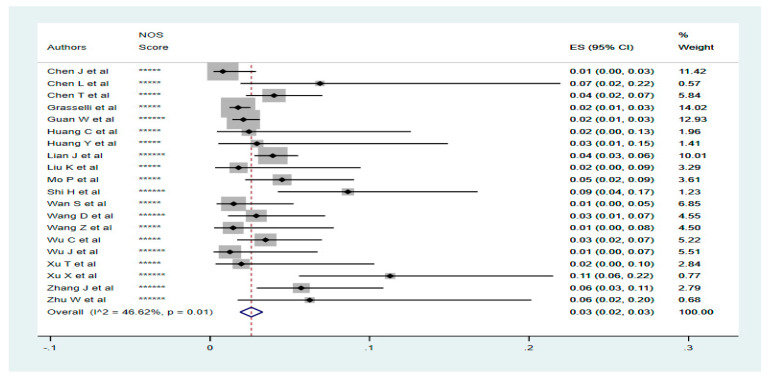
Pooled prevalence of patients with liver diseases (Chronic Liver Diseases, Hepatitis B/C infections) diagnosed with COVID-19. The red dotted line represents the overall effect size of the studies (0.03). The edges of the blue diamond represent 95% confidence intervals (0.02, 0.03). ES = Effect Size, NOS = Newcastle-Ottawa Score.

The prevalence of liver diseases in patients diagnosed with COVID-19 is 3% (95% CI; 2–3%). A random effect model was used for pooling the studies because of the observed low level of between-studies heterogeneity (I^2^ = 46.62%, *p* = 0.01) (Figure 3).

### 3.5. Disease Outcome for Liver Diseases Patients with COVID-19

Six of the 22 included studies reported severity in COVID-19 patients with different forms of liver disease. The 6 studies including 3182 COVID-19 patients, 75 of which had underlying liver diseases, reported severity in 57.33% (43/75) of cases. Two of the 22 studies reported mortality in COVID-19 patients with liver diseases. These studies included 1373 COVID-19 cases, 34 of which had liver diseases at the time of diagnosis. In all, 6 deaths (17.65%) were recorded.

## 4. Discussion

We report here, for the first time, the prevalence, severity and mortality of patients diagnosed with COVID-19 with underlying chronic kidney disease and liver diseases. Our outcomes show that the overall prevalence of CKD and Liver Diseases in COVID-19 are 1% and 3% respectively. We also report a COVID-19 severity of 83.93% (47/56) in patients with CKD and 57.33% (43/75) in patients with liver diseases. The rate of mortality in COVID-19 patients with CKD and Liver diseases was found to be 53.33% (8/15) and 17.65% (6/34) respectively.

The presence of comorbidities is associated with poor prognoses in patients with COVID-19, with higher mortality rates and severity. The most common comorbidities reported so far in severe cases have been hypertension, diabetes, cardiovascular diseases, cerebrovascular diseases and COPD [29]. However, how these diseases contribute to the COVID-19 outcome remains unclear. 

Biomarkers of liver injuries have been reported to increase in patients with COVID-19 [25,35,45], although, no virus was found in the liver tissue of patients who died from the disease [54]. This is to be expected, as angiotensin II-converting enzyme (ACE2) receptor, a key player in the “docking” and replication of the SARS-CoV-2 virus, is not expressed in hepatocytes. However, ACE2 expression has been reported in cholangiocytes [55], leading to the suggestion that the binding of SARS-CoV-2 to the epithelial cells of the biliary tree may cause biliary dysfunction [56]. Zhang et al. also suggested that the transient liver injuries observed in COVID-19 patients may be associated with drug toxicity, cytokine storm or hypoxia [56]. While many studies have reported liver dysfunction in COVID-19 [25,35,45], the mechanistic link between the two remains to be established. 

Furthermore, CKD have been associated with inflammation and dysregulation of the immune system [57]. This dysregulation of immune function, which may exist in patients with underlying CKD, may explain the increased severity and mortality due to COVID-19. The levels of ACE2 receptor in the kidney have been previously reported to be altered in patients with human kidney diseases [58]. In a recent study by Fan et al., it was reported that ACE2 receptor is overexpressed in the tubular cells of patients with CKD. Alteration in kidney functions, characterised by increased serum creatinine and urea nitrogen, was also reported in patients with COVID-19 [59]. Taken together, the alterations in ACE2 receptor expression may explain the observed kidney dysfunction in COVID-19 and provide the answer to why patients with CKD are vulnerable to the SARS-CoV-2 virus. 

This study is limited by several factors. Firstly, some included studies did not report comorbidities. Where comorbidities were specified, the criteria for defining severity were not uniform. Some studies included only patients with primary composite outcomes, while some did not report mortality. Lastly, the aetiology and pathophysiological characteristics of the comorbidities were not documented. 

Indeed, this review involved an in-depth literature search followed by a systematic analysis of data involving a total of 5595 patients with confirmed COVID-19. For the first time, we have established the potential risk of COVID-19 in liver disease and CKD patients, which indicates an increased vulnerability of this subpopulation. 

The most important clinical implication of this study is that Liver disease and CKD patients are potentially highly vulnerable to COVID-19 and should be considered for remote consultation and the most stringent social isolation to prevent infection. Future studies should investigate how liver diseases and CKD contribute to poor prognoses in COVID-19.

## 5. Conclusions

We report a potential increased risk of severity and mortality in COVID-19 patients with liver diseases and CKD. This study will facilitate better clinical management and inform the process of implementing more stringent preventative measures for this group of patients. 

## Figures and Tables

**Table 1:** Characteristics of the studies included. Severity is defined as extended hospital stay, need for ICU or mechanical ventilation. Mortality is defined as death associated with COVID-19.

Authors	Country	Study Type	Sample Size	Female (%)	Mortality (%)	Follow-up Time (Days)	Liver Patients (%)	Liver Severity (%)	Liver Deaths (%)	Renal Patients (%)	Renal Severity (%)	Renal Death (%)
Chen J et al. [33]	China-Shanghai	Retrospective Analysis	249	123/249 (49.40)	2/249 (0.80)	16	2/249 (0.80)	-	-	-	-	-
Chen L et al. [34]	China-Wuhan	Retrospective Analysis	29	8/29 (27.59)	26/29 (89.66)	15	2/29 (6.90)	-	-	-	-	-
Chen N et al. [35]	China-Wuhan	Descriptive Analysis	99	32/99 (32.32)	11/99 (11.11)	20	-	-	-	3/99 (3.03)	-	-
Chen T et al. [36]	China-Wuhan	Retrospective Analysis	274	103/274 (37.59)	113/274 (41.24)	30	11/274 (4.02)	-	5/11 (45.46)	5/274 (1.83)	-	4/5 (80.00)
Grasselli et al. [37]	Italy-Multicentre	Retrospective Analysis	1591	287/1591 (18.04)	405/1591 (25.46)	34	28/1591 (1.76)	28/28 (100.00)	-	36/1591 (2.26)	36/36 (100.00)	-
Guan W et al. [38]	China-Multicentre	Retrospective Analysis	1099	459/1099 (41.77)	15/1099 (1.37)	49	23/1099 (2.09)	1/23 (4.35)	1/23 (4.35)	8/1099 (0.73)	3/8 (37.50)	2/8 (25.00)
Huang C et al. [25]	China-Wuhan	Prospective Analysis	41	11/41 (26.83)	-	32	1/41 (2.44)	-	-	-	-	-
Huang Y et al. [39]	China-Wuhan	Retrospective Analysis	34	20/34 (58.82)	-	39	1/34 (2.94)	-	-	-	-	-
Lian J et al. [40]	China-Zhejiang	Retrospective Analysis	788	381/788 (48.35)	-	26	31/788 (3.93)	-	-	7/788 (0.89)	-	-
Liu K et al. [41]	China-Hainan	Retrospective Analysis	56	25/56 (44.64)	3/56 (5.36)	46	1/56 (1.79)	-	-	1/56 (1.79)	-	-
Mo P et al. [42]	China-Wuhan	Retrospective Analysis	155	69//155 (44.52)	22/155 (14.19)	36	7/155 (4.52)	5/7 (71.43)	-	6/155 (3.87)	4/6 (66.67)	-
Shi H et al. [43]	China-Wuhan	Descriptive Analysis	81	39/81 (48.15)	3/81 (3.70)	47	7/81 (8.64)	-	-	3/81 (3.70)	-	-
Wan S et al. [44]	China-Chongqing	Retrospective Analysis	135	63/135 (46.67)	1/135 (0.74)	16	2/135 (1.48)	1/2 (50.00)	-	-	-	-
Wang D et al. [45]	China-Wuhan	Retrospective Analysis	138	63/138 (45.65)	6/138 (4.35)	34	4/138 (2.90)	-	-	4/138 (2.90)	2/4 (50.00)	-
Wang Z et al. [46]	China-Wuhan	Retrospective Analysis	69	37/69 (53.62)	5/69 (7.25)	19	1/69 (1.45)	-	-	-	-	-
Wu C et al. [47]	China-Wuhan	Retrospective Analysis	201	73/201 (36.32)	44/201 (21.89)	63	7/201 (3.48)	-	-	2/201 (1.00)	-	-
Wu J et al. [48]	China-Jiangsu	Retrospective Analysis	80	41/80 (51.25)	-	23	1/80 (1.25)	-	-	1/80 (1.25)	-	-
Xu T et al. [49]	China-Changzhou	Retrospective Analysis	51	26/51 (50.98)	-	35	1/51 (1.96)	-	-	1/51 (1.96)	-	-
Xu X et al. [50]	China-Zhejiang	Retrospective Analysis	62	27/62 (43.55)	-	16	7/62 (11.29)	4/7 (57.14)	-	1/62 (1.61)	-	-
Zhang J et al. [51]	China-Wuhan	Retrospective Analysis	140	69/140 (49.29)	-	34	8/140 (5.71)	4/8 (50.00)	-	2/140 (1.43)	2/2 (100.00)	-
Zhou et al. [52]	China-Wuhan	Retrospective Analysis	191	72/191 (37.70)	54/191 (28.27)	15	-	-	-	2/191 (1.05)	-	2/2 (100.00)
Zhu W et al. [53]	China-Anhui	Retrospective Analysis	32	17/32 (53.13)	-	27	2/32 (6.3)	-	-	1/32 (3.13)	-	-
**Total**			5595	2045/5595 (36.55)	710/4367 (16.26)	672	147/5305 (2.77)	43/75 (57.33)	6/34 (17.65)	83/5038 (1.65)	47/56 (83.93)	8/15 (53.33)

**Figure A1:**
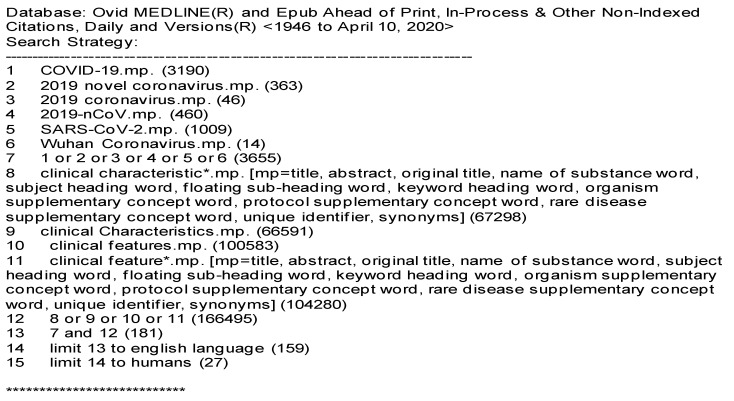
Medline Search Strategy.

**Figure A2:**
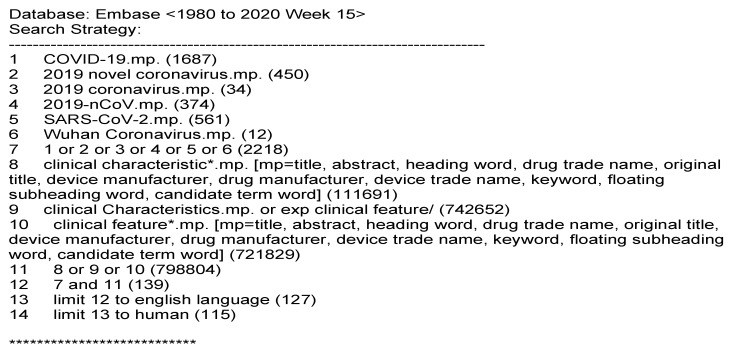
Embase Search Strategy.

## Appendix A

**Table A1:** Quality Assessment.

First Author	SELECTION		ASCERTAINMENT		OUTCOME		OVERALL
	Sample Size Adequate	Population Representative (Multicentre)	Test Adequate	Comorbidity Confirmation Adequate (EMR)	Outcome Reported Adequately (Clinical Staff)	Follow-up Long Enough (≥2 Weeks)	OVERALL (≥4 Stars = Lower Risk of Bias)
Chen J et al.	*		*	*	*	*	*****
Chen L et al.	*		*	*	*	*	*****
Chen N et al.	*		*	*	*	*	*****
Chen T et al.	*		*	*	*	*	*****
Grasselli et al.	*	*	*		*	*	*****
Guan W et al.	*	*	*	*	*	*	******
Huang C et al.	*		*	*	*	*	*****
Huang Y et al.	*		*	*	*	*	*****
Lian J et al.	*	*	*	*	*	*	******
Liu K et al.	*		*	*	*	*	*****
Mo P et al.	*		*	*	*	*	*****
Shi H et al.	*	*	*	*	*	*	******
Wan S et al.	*		*	*	*	*	*****
Wang D et al.	*	*	*	*	*	*	******
Wang Z et al.	*		*	*	*	*	*****
Wu C et al.	*		*	*	*	*	*****
Wu J et al.	*	*	*	*	*	*	******
Xu T et al.	*		*	*	*	*	*****
Xu X et al.	*	*	*	*	*	*	******
Zhang J et al.	*		*	*	*	*	*****
Zhou et al.	*	*	*	*	*	*	******
Zhu W et al.	*	*	*	*	*	*	******
